# Impacts of global warming on residential heating and cooling degree-days in the United States

**DOI:** 10.1038/srep12427

**Published:** 2015-08-04

**Authors:** Yana Petri, Ken Caldeira

**Affiliations:** 1 Carnegie Institution for Science, Department of Global Ecology, Stanford, CA, USA

## Abstract

Climate change is expected to decrease heating demand and increase cooling demand for buildings and affect outdoor thermal comfort. Here, we project changes in residential heating degree-days (HDD) and cooling degree-days (CDD) for the historical (1981–2010) and future (2080–2099) periods in the United States using median results from the Climate Model Intercomparison Project phase 5 (CMIP5) simulations under the Representation Concentration Pathway 8.5 (RCP8.5) scenario. We project future HDD and CDD values by adding CMIP5 projected changes to values based on historical observations of US climate. The sum HDD + CDD is an indicator of locations that are thermally comfortable, with low heating and cooling demand. By the end of the century, station median HDD + CDD will be reduced in the contiguous US, decreasing in the North and increasing in the South. Under the unmitigated RCP8.5 scenario, by the end of this century, in terms of HDD and CDD values considered separately, future New York, NY, is anticipated to become more like present Oklahoma City, OK; Denver, CO, becomes more like Raleigh, NC, and Seattle, WA, becomes more like San Jose, CA. These results serve as an indicator of projected climate change and can help inform decision-making.

In 2009, according to the estimates from the Residential Energy Consumption Survey (RECS), space heating and air conditioning accounted for 41% of average US household end-use energy expenditures^1^. As fluctuations in outside air-temperature are known to have a substantial impact on weather-related building energy use^2^,^3^ and thus a substantial influence on the expenses of residents^4^, it is of interest to estimate the future effects of climate change on the magnitude and spatial patterns of heating and cooling demand in various US regions. For instance, a recent synthesis report^5^ released by the US Climate Change Science Program evaluated approximately 20 assessments of US building energy sector sensitivity to global warming published since 1990, concluding that the total projected average across the surveyed national studies is a 9.2% energy reduction for heating and a 5–20% increase for cooling per 1 °C of temperature increase. Since global mean surface temperature is expected to rise by 2.6–4.8 °C by year 2100 under the RCP8.5 unmitigated high emission scenaro^6^, climate change is projected to alter the present balance between residential heating and cooling needs across the country, potentially causing regions with moderate climates to warm and shift some locations from primarily heating to primarily cooling demand^4^,^5^,^7^,^8^.

Previous studies used numerous methods for estimating residential heating and cooling demand, including building^5^ and climate^8^ simulation models, econometrics^5^, and statistical analyses^5^. In addition, many publications employed degree-days, which are defined as integrated temperature deviations from a base temperature over time^9^. The base temperature is a point at which internal gains (e.g. heat from occupants, lights, equipment, etc.)^10^ equal the heat loss, or a threshold below (or above) which heating (or cooling) appliances do not need to operate in order to maintain indoor thermal comfort^9^. Robust degree-day formulas require^9^ the knowledge of a building’s unique base temperature, calculated from its heat loss coefficient, thermal capacity, and infiltration. These factors, however, are not taken into account if the standard US base temperature^8^,^9^,^10^,^11^ of 65 °F (18.3 °C) is used. The most precise way to determine degree-days is to use the mean degree-hours method^9^; yet, if the weather data is limited to maximum and minimum daily temperatures, either the mean daily temperature approach^9^ or the Meteorological Office equations^9^ could be used, which give a lower per cent error^9^ (see Methods). Notably, even though the degree-day technique is associated with some limitations^9^,^12^, it accurately captures the duration and severity of weather events^9^,^13^, thus becoming a useful tool for evaluating residential heating and cooling demand, as well as outdoor thermal comfort. Here, we focus only on heating and cooling degree-days, and do not evaluate the associated energy requirements or costs. Assuming that they are mathematically defined in an internally consistent way, metrics such as HDD and CDD cannot be right or wrong; they can be more or less useful. We suggest that HDD and CDD values are useful in helping to understand the magnitude^8^ of climate change projected for the United States.

In the last 20 years, US heating degree-days (HDD) declined and cooling degree-days (CDD) increased due to warming trends^5^,^7^,^14^, suggesting an overall spatial redistribution of residential heating and cooling demand and expenditures, as well as outdoor thermal comfort. Preceding investigators^15^,^16^,^17^,^18^ examined the historical allocation of HDD and CDD separately, but Sivak^10^,^19^ proposed summing HDD and CDD to produce a combined degree-day index which could be a valuable consideration in the process of deciding where to live in the United States. Equally weighing HDD and CDD does not consider^10^ the distinctions in human perception of heat and cold, types of building insulation, appliances’ efficiency, and weather-related energy expenditures^4^. Nonetheless, the unweighted sum of HDD and CDD has previously been applied as an easy-to-understand indication of differences in heating and cooling demand^10^,^19^ to provide an approximate comparative measure of outdoor thermal comfort at various locations in the US. Therefore, understanding the limitations of this approach, we adopt Sivak’s combined HDD and CDD metric^10^,^19^ here and designate it with the symbol “HDD + CDD”. Previous literature on future degree-day scenarios in context of global warming did not investigate HDD + CDD, however, it has come to several important conclusions regarding HDD and CDD considered separately. Firstly, HDD were projected to decrease, while CDD were expected to grow by the late century, both countrywide^5^,^7^,^18^,^20^,^21^,^22^,^23^,^24^,^25^ and worldwide^17^,^24^,^26^,^27^,^28^, albeit the rate and magnitude of change in each parameter differed with the study. Secondly, changes in national residential net use of delivered energy were anticipated to result in modest savings^5^,^20^,^21^,^22^,^26^, because the reduction in heating fuels was expected to more than offset the rise in electricity demand for air conditioning^5^. However, on a regional level, combined energy demand was forecasted to exhibit spatial heterogeneity^20^: In northern areas with more than 4,000 HDD, the combined degree-day index dropped substantially, as compared to the areas in the South, where a reverse climatic effect was anticipated^5^.

US policymakers and utility regulators could benefit^20^,^29^ from considering the conclusions of the studies discussed above. Nonetheless, residents remain largely unassisted in the selection of housing locations based on key indicators of future outdoor thermal comfort and combined heating and cooling demand. Focusing on investment strategies^4^ and energy planning^20^, previous works employed population-weighted HDD and CDD, which classified territories according to the total required energy, as opposed to per-person weather related energy use. Earlier research usually addressed temperature-dependent energy demand on a national, state, or city scale, computing net area averages, instead of providing region-specific details.

Here, we elaborate on the formerly mentioned limitations and suggest that HDD + CDD could provide a key indicator not only of heating and cooling demand in residential US buildings, but also of outdoor thermal comfort. For example, because the standard US base temperature of 65 °F (18.3 °C) used in this study for both cooling and heating degree-days is within the narrow temperature range considered comfortable by most people^9^,^30^, regions with a smaller three-decade sum of outdoor temperature deviations from this base temperature will be more likely to have comfortable outdoor thermal conditions. We recognize that factors such as relative humidity, solar insulation, and wind play an important role in determining outdoor thermal comfort. However, these considerations are outside the scope of this study.

In this work, using the latest US Climate Normals developed by the National Oceanic and Atmospheric Administration (NOAA)^31^,^32^, along with state-of-the-art CMIP5 multi-model ensemble simulations^33^, we find areas with the maximum and minimum combined degree-day sum for the historical (1981–2010) and future (2080–2099) periods under the unmitigated RCP8.5 climate scenario.

## Results

### Historical (1981–2010) 30-year annual US degree-days

**Figure 1:**
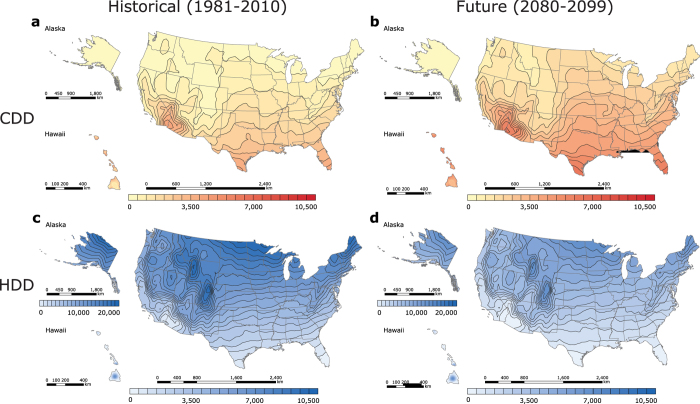
Maps of interpolated historical (1981–2010) and future (2080–2099) US CDD and HDD, calculated from NOAA’s 30-year daily degree-day normals and CMIP5 RCP8.5 climate model output, respectively. (**a**) Historical (1981**–**2010) CDD. (**b**) Future (2080–2099) CDD. (**c**) Historical (1981**–**2010) HDD. (**d**) Future (2080–2099) HDD. Historical CDD and HDD were computed from temperature data recorded by 7,438 US weather stations. In Fig. 1c,d, Alaska (0–20,000 HDD, step 1,000) is found on a different scale than the contiguous US and Hawaii (0–10,500 HDD, step 500), because of Alaska’s large HDD values. The maps were generated in ESRI ArcMap 10.2 (Environmental Systems Resource Institute, ArcMap 10.2 ESRI, Redlands, California, USA) using an ordinary kriging tool.

NOAA’s 30-year (1981–2010) US degree-day normals^31^,^32^, calculated for 7,438 weather stations, have CDD values that differ substantially among different regions of the US. When the station data is interpolated to maps, the interpolated CDD values indicate degree-day estimates for locations that lack weather stations (Fig. 1a). The contiguous US is distinguished by a large spread in CDD values, which generally increase from the North to the South^5^,^20^ and decrease at high latitudes. Places in the contiguous US that have the least cooling demand are dispersed across the North of the country and include the Rocky Mountains (with values ranging from 0 to 500 CDD). Conversely, sites that demand the most cooling are approximately located at (1) the intersection of California and Arizona, (2) Southern Texas, McAllen region, and (3) Southern Florida, Miami area (4,000 to 4,500 CDD). To characterize CDD within larger regions, we refer to the station median values within those regions. This approach indicates that the overall highest (3,331 CDD) and lowest (3 CDD) CDD medians are found in climatically extreme Hawaii and Alaska, respectively, while an intermediate CDD value (822 CDD) is estimated for the contiguous US. The hottest regions in the contiguous US substantially differ from the areas in Alaska, where all CDD values are close to 0.

Across the US, HDD values usually vary inversely with CDD values: areas with high HDD values correspond to areas with low CDD values, and vice versa. This is evident in the north-south HDD decrease across the contiguous US^5^,^20^ (Fig. 1c). As another example, locations that have the lowest HDD values in the contiguous US, such as Southern Florida, the area of Miami (0 to 500 HDD), have the greatest CDD values. On the contrary, regions that have the highest HDD values, such as (1) western Wyoming, (2) upper North Dakota, and (3) northern Minnesota (10,000 to 10,500 HDD), have the lowest CDD values. Nationally, HDD values tend to exceed^5^,^20^,^24^ CDD values. For example, median HDD values in the contiguous US (5,514 HDD) and Alaska (10,958 HDD) exceed corresponding median CDD values by multiplicative factors of 7 and 3,321, respectively. The Hawaii HDD median, however, is only equivalent to 1, because the state is located in the warm tropics.

**Figure 2:**
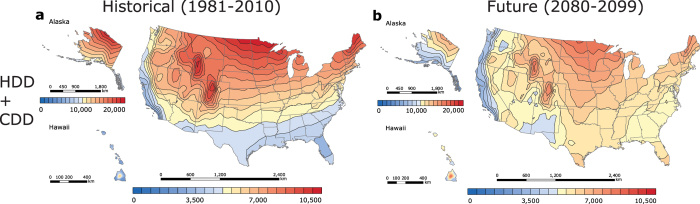
Maps of interpolated historical (1981–2010) and future (2080–2099) US HDD + CDD, calculated from NOAA’s 30-year daily degree-day normals and CMIP5 RCP8.5 climate model output, respectively. (**a**) Historical (1981**–**2010) HDD + CDD. (**b**) Future (2080–2099) HDD + CDD. Historical HDD + CDD were computed from temperature data recorded by 7,438 US weather stations. In Fig. 2, Alaska (0–20,000 HDD + CDD, step 1,000) is found on a different scale than the contiguous US and Hawaii (0–10,500 HDD + CDD, step 500), because of Alaska’s large HDD + CDD values. The maps were generated in ESRI ArcMap 10.2 (Environmental Systems Resource Institute, ArcMap 10.2 ESRI, Redlands, California, USA) using an ordinary kriging tool.

To provide a key indicator of the total heating and cooling demand and outdoor thermal comfort at various US locations, we add HDD to CDD, producing a combined degree-day metric^10^,^19^. Other key indicators could be based on the amount of sunshine, humidity, wind, and characteristics of the built environment. A map based on this interpolated HDD + CDD sum is illustrated (Fig. 2a). In the US, HDD + CDD values are shown to be largest in the northern regions, where the highest HDD values are greater than the highest CDD values in all other areas. Because the number of HDD in the contiguous US tends to predominate over the number of CDD, HDD + CDD values typically decrease from the North to the South, but exhibit a less homogenous trend than HDD values. Areas with the lowest degree-day sum in the contiguous US are located in the West coast of California (1,875 to 2,500 HDD + CDD). In contrast, locations with the greatest degree-day sum are (1) western Wyoming, (2) upper North Dakota, and (3) northern Minnesota (10,000 to 10,500 HDD + CDD), which are also the regions with the largest HDD values. The correlation between high HDD values and high HDD + CDD values is evidenced by the relationship between the respective station median degree-days. Compared to the contiguous US (6,386 HDD + CDD) and Hawaii (3,439 HDD + CDD), Alaska, where HDD predominate, has the largest median degree-day sum (10,959 HDD + CDD). Overall, in the United States, HDD + CDD values tend to increase with substantial rises in elevation. This trend is evident in Wyoming and Colorado, where the highest points (Gannett Peak and Mount Elbert, respectively) are located in the same regions as the highest HDD + CDD values. For example, Denver, CO, has a larger (6,671 HDD + CDD) median degree-day sum than Nashville, TN, (5,350 HDD + CDD), which is found at a lower elevation.

**Figure 3:**
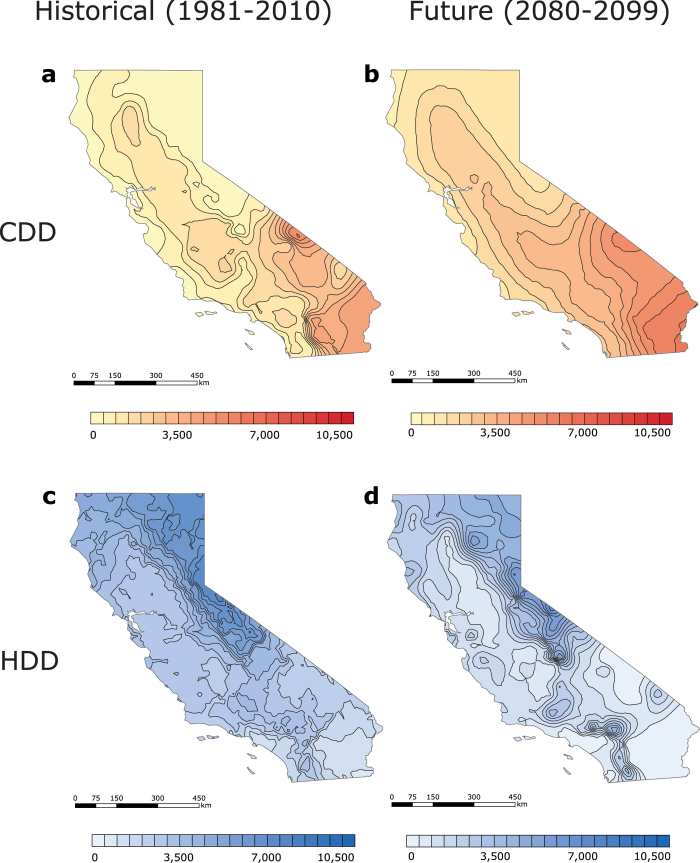
Maps of California’s historical (1981–2010) and future (2080–2099) US CDD and HDD, calculated from NOAA’s 30-year daily degree-day normals and CMIP5 RCP8.5 climate model output, respectively. (**a**) Historical (1981**–**2010) CDD. (**b**) Future (2080–2099) CDD. (**c**) Historical (1981**–**2010) HDD. (**d**) Future (2080–2099) HDD. Historical CDD and HDD were computed from temperature data recorded by 392 US weather stations. The maps were generated in ESRI ArcMap 10.2 (Environmental Systems Resource Institute, ArcMap 10.2 ESRI, Redlands, California, USA) using an ordinary kriging tool.

**Figure 4:**
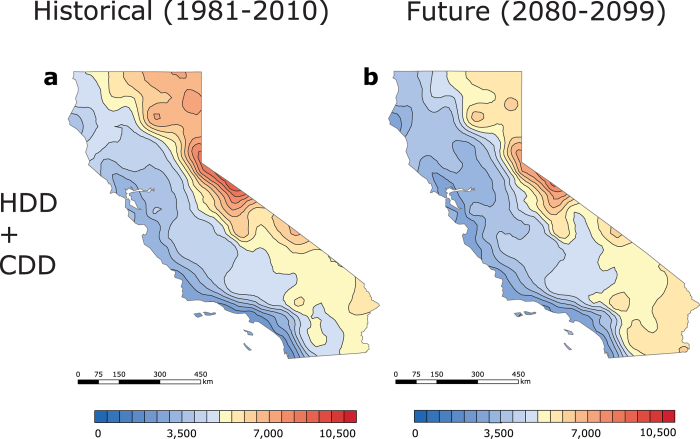
Maps of California’s historical (1981–2010) and future (2080–2099) US HDD + CDD, calculated from NOAA’s 30-year daily degree-day normals and CMIP5 RCP8.5 climate model output, respectively. (**a**) Historical (1981**–**2010) HDD + CDD. (**b**) Future (2080–2099) HDD + CDD. Historical HDD + CDD were computed from temperature data recorded by 392 US weather stations. The maps were generated in ESRI ArcMap 10.2 (Environmental Systems Resource Institute, ArcMap 10.2 ESRI, Redlands, California, USA) using an ordinary kriging tool.

Because California encompasses regions with the lowest degree-day sum in the contiguous US, the degree-days of this state are plotted at a finer scale and investigated separately (Figs 3a,c and 4a). The low HDD + CDD values (Fig. 4a) could be attributed to the favourable balance between heating and cooling demand in California, as well as its geographical location. The California CDD station median (846 CDD) is approximately equal to the national CDD station median. CDD values are higher in the hot California’s deserts and in the Central Valley, but are lower along the Pacific coast (Fig. 3a). Mild areas that surround the northern part of the Central Valley (i.e. Del Norte, Siskiyou, Modoc, etc.), as well as California’s coastal zones (i.e. San Francisco, San Mateo, Monterey, etc.) have the lowest CDD values (0 to 625 CDD), while Death Valley in Inyo County has the highest CDD values (5,000 to 5,625 CDD). The California HDD station median (2,666 HDD), however, is about half as small as the national HDD station median, which accounts for the state’s low net heating and cooling demand. In California, only the regions near the Cascade Mountains and Sierra Nevada require substantial amounts of heating (Fig. 3c). Locations with the smallest HDD values are found in eastern San Bernardino, Riverside, and Imperial counties (625 to 1,250 HDD), whereas areas with the greatest HDD values are situated on the intersection of Alpine and Mono counties (7,500 to 8,125 HDD). Although Hawaii has the lowest historical station median degree-day sum in the nation, California has the lowest historical degree-day sum (4,180 HDD + CDD) in the contiguous US (Fig. 4a). Coastal areas of Ventura, Los Angeles, Orange, and San Diego counties have the lowest^8^ degree-day sum (1,875 to 2,500 HDD + CDD), and areas within the northern Mono County have the highest degree-day sum (8,750 to 9,375 HDD + CDD).

### Future (2080–2099) 30-year annual US degree-days

**Figure 5:**
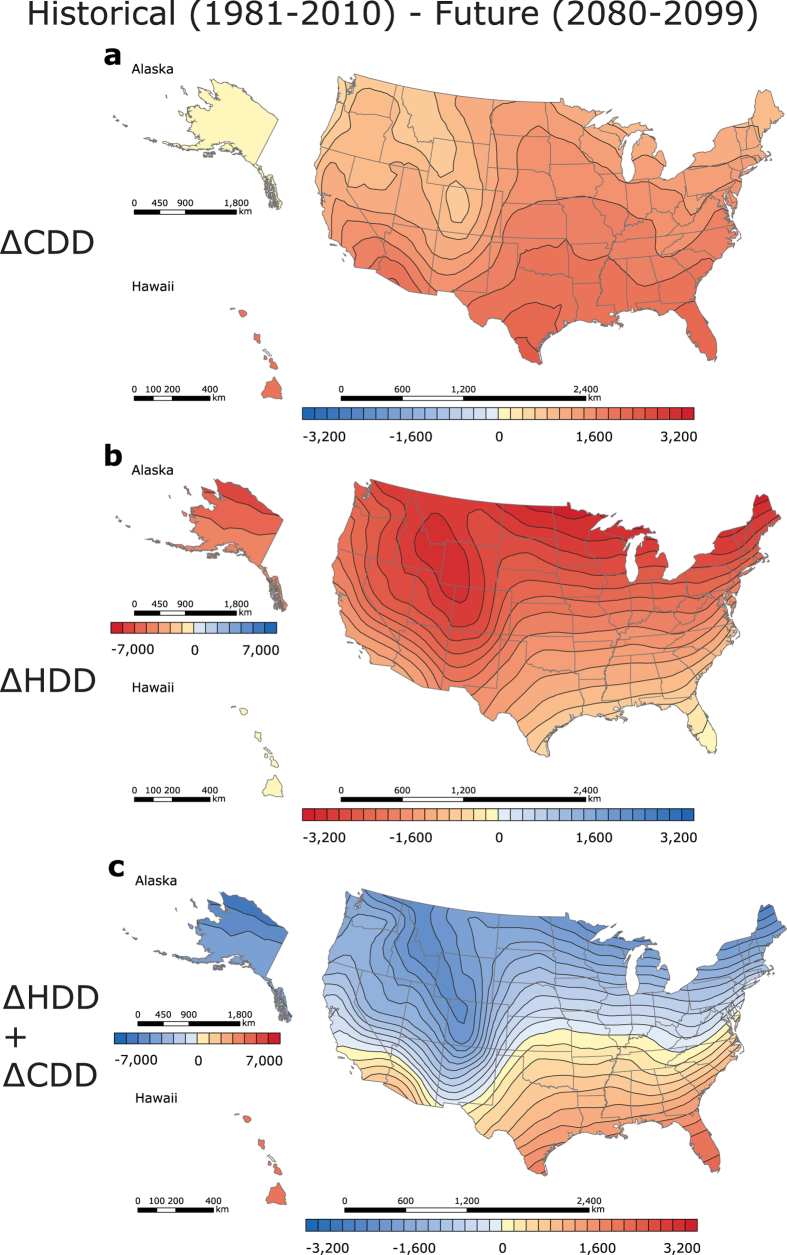
CMIP5 future (2080–2099) changes in historical (1981–2010) US CDD, HDD, and HDD + CDD. (**a**) ΔCDD. (**b**) ΔHDD. (**c**) ΔHDD + ΔCDD. In Fig. 5b,c, Alaska (−7,000–7,000 Δ degree-days, step 1,000) is found on a different scale than the contiguous US and Hawaii (−3,200–3,200 Δ degree-days, step 200), because of Alaska’s high Δ degree-day values. Δ Degree-days were obtained by subtracting historical (1981–2010) annual degree-days (calculated for each NOAA’s weather station) from the CMIP5 multi-model median degree-day projections (2080–2099). The maps were generated in ESRI ArcMap 10.2 (Environmental Systems Resource Institute, ArcMap 10.2 ESRI, Redlands, California, USA) using an ordinary kriging tool.

Analysing the historical (1981–2010) distribution of annual US degree-day normals^31^,^32^, we use median projections from the CMIP5 multi-model ensemble to estimate end-of-century (2080–2099) heating and cooling demand under the “business-as-usual” RCP8.5 high emission climate scenario^34^ (see Methods). Our results demonstrate that the spatial pattern of future (2080–2099) degree-day values is expected to alter somewhat at national scale; however, at regional scale, substantial changes are apparent (Figs 1b,d, 2b, and 5). In the contiguous US, for example, the usual trend in the north-south increase in CDD values is evident (Fig. 5a). However, the map’s isolines move up, thus indicating increased cooling demand in regions with historically favourable CDD values^5^,^20^,^24^. The region with the lowest CDD values will shift northward and be located in coastal Washington, Oregon, and the northern part of the Rocky Mountains (500 to 1,000 CDD). The geographic areas with the highest CDD values will remain similar over time, but the CDD values in these areas increase from 6,000 CDD in the 1981–2010 period to 6,500 CDD in the 2080–2099 period. Notably, North-western Washington will have the lowest increase in CDD values (+300 to 450 ΔCDD), while Southern Texas will have the greatest increase in CDD values (+2,250 to 2,400 ΔCDD) (Fig. 5a). As the result of rising temperatures, median cooling demand can be expected to grow in all regions; new station CDD medians estimated to be 2,215 CDD for the contiguous US, 91 CDD for Alaska, and 5,288 CDD for Hawaii. Areas with high historical CDD values (e.g. Hawaii, +1,962 ΔCDD) will experience a substantial rise in CDD values, while areas with intermediate or low historical CDD values (e.g. the contiguous US, +1,423 ΔCDD; Alaska, +84 ΔCDD) will experience a moderate increase in CDD values.

In the contiguous US, the trend in the north-south HDD decrease remains present, but the map’s isolines (Fig. 1d) shift up, thus indicating the potential for reduced heating demand in southern residential buildings^23^. In addition to the historically warm Central and Southern Florida, for example, Southern Texas, as well as California and Arizona intersection, are expected to have the smallest heating demand (0 to 500 HDD) in the US. The northern Minnesota border and the central Rocky Mountains region in Wyoming, on the contrary, are expected to have the greatest heating demand (7,000 to 7,500 HDD). Furthermore, Southern Florida is projected to experience the smallest decrease in HDD values (−150 to 0 HDD), whilst upper North Dakota, Minnesota, and Maine are anticipated to have the largest decrease in HDD values (−3,150 to 3,000 HDD). The reduction in station median HDD is projected to exceed the growth in CDD in the contiguous US (3,470 HDD; −2,011 ΔHDD) and Alaska (7,403 HDD; −3,504 ΔHDD) (Figs 1d and 5b). In the state of the largest HDD decrease, Alaska, the lowest HDD values are anticipated in the Southeast (4,000 to 5,000 HDD), and the highest HDD values are expected in the Far North (13,000 to 14,000 HDD). Yet, in Hawaii (1 HDD, 0 ΔHDD), the station CDD median is projected to exceed the station HDD median.

Interestingly, a new trend will emerge in the contiguous US at the end of the century (Fig. 5с): ΔHDD + ΔCDD will decrease in the North and increase in the South, creating an invisible “0 change” line that will divide the country into two approximately equal regions. Along this line, decreases in HDD will be balanced by increases in CDD. The national distribution of HDD + CDD is expected to be heterogeneous^20^, and is thus not anticipated to decline in the north-south direction (Fig. 2b). The California coast is expected to have the lowest degree-day sum (2,500 to 3,000 HDD + CDD) in the 50 United States, and upper North Dakota is expected to have the highest degree-day sum (8,500 to 9,000 HDD + CDD) in the contiguous US. The largest net degree-day reduction is projected to occur in northern Maine (−2,550 to −2,400 ΔHDD + ΔCDD), while the greatest increase in the degree-day sum is anticipated to occur in Southern Florida (+1,950 to 2,100 ΔHDD + ΔCDD) (Fig. 5c). Furthermore, as a result of anthropogenic climate change, station median degree-day sums in the contiguous US (5,872 HDD + CDD; −614 ΔHDD + ΔCDD) and Alaska (7,518 HDD + CDD; −3,589 ΔHDD + ΔCDD) are projected to decrease, with a reverse effect observed in Hawaii (5,408 HDD + CDD; +1,961 ΔHDD + ΔCDD) (Figs 2b and 5с). The mild Alaskan Southeast is projected to have the smallest degree-day sum (4,000 to 5,000 HDD + CDD), while the arctic Far North is anticipated to have the greatest degree-day sum (13,000 to 14,000 HDD + CDD). In Hawaii, the lowest degree-day sum will predominate on the shores (3,500 to 4,000 HDD + CDD), and the highest combined heating and cooling demand will occur in the Mauna Loa region (8,000 to 8,500 HDD + CDD).

Because the historical degree-day sum in Hawaii is projected to increase more rapidly than in other US regions, California, where annual HDD + CDD normals decline with time, is in the future (2080–2099) projected to achieve the lowest national combined demand for heating and cooling, which deserves a closer examination (Figs 3b,d and 4b). By the end of the century, California’s station median CDD values are anticipated to rise (2,139 CDD; +1,200 ΔCDD), while places with the lowest historical CDD values (625 to 1,250 CDD) are expected to decrease in area, shifting further north (Fig. 3b). The locations with the highest CDD (5,625 to 6,250 CDD) will be found in eastern San Bernardino, Riverside, and Imperial. Notably, Death Valley will no longer be located in the region with the greatest number of CDD. The lowest increase in CDD values will occur in western Del Norte (+600 to 650 ΔCDD), and the greatest growth in CDD values will occur in eastern Imperial (+1,850 to 1,900 ΔCDD). California’s station median HDD will decrease from the South to the North (Fig. 3d). Counties within Southern California (i.e. Inyo, San Bernardino, Los Angeles, etc.) are projected to have the lowest HDD values (0 to 625 HDD), while eastern Mono (6,875 to 7,500 HDD) is expected to have the greatest HDD values. Eastern Modoc is projected to experience the smallest change in HDD values (−1,000 to −950 ΔHDD), while north-western San Diego is anticipated to experience the greatest change in HDD values (−2,350 to −2,300 ΔHDD). California station median HDD + CDD is expected to decrease (3,752 HDD + CDD; −345 ΔHDD + ΔCDD) relative to the historical HDD + CDD values (Fig. 4b). Coastal Ventura County will have the lowest degree-day sum (1,875 to 2,500 HDD + CDD) in the state; Mono County, in contrast, is projected to have the highest degree-day sum (7,500 to 8,125 HDD + CDD). Similarly to the contiguous US, California will be divided into two distinct regions: the “North”, where HDD + CDD is anticipated to decrease, and the “South”, where HDD + CDD is projected increase. The largest decline in the degree-day sum will occur in eastern Modoc County (−500 to −450 ΔHDD + ΔCDD), while the greatest increase in HDD + CDD will occur in Southern Imperial (+50 to 100 ΔHDD + ΔCDD).

### Historical (1981–2010) and future (2080–2099) degree-days in 25 US cities

We tabulate historical (1981–2010) and projected (2080–2099) annual HDD, CDD, and HDD + CDD normals interpolated to locations of 25 cities chosen from a list of the 50 most populous incorporated places in the US^35^ (Table 1; for information on the historical (1981–2010), projected (2080–2099), and Δ degree-days in all of the 50 most populous US cities, see Supplementary Tables S2 and S3). These cities were chosen to illustrate different climate regimes and emphasize geographic diversity. Among these cities, in the historical period (1981–2010), San Francisco, CA, has the lowest CDD value (163 CDD), while Phoenix, AZ, has the highest CDD value (4,608 CDD). Miami, FL, on the other hand, has the lowest historical HDD value (128 HDD), and Minneapolis, MN, has the highest historical HDD value (7,581 HDD). As the combined heating and cooling demand tends to be dominated by heating^5^, the HDD + CDD metric tends to be dominated by HDD. Consistent with this observation, the city of San Diego, CA, is estimated to have the smallest historical degree-day sum (1,946 HDD + CDD), and Minneapolis, MN, is expected to have the greatest historical degree-day sum (8,333 HDD + CDD). Based on projections of future (2080–2099) US climate, Minneapolis, MN, will continue to have the greatest degree-day sum (7,174 HDD + CDD) at the end of the century; however, in the same future period, San Francisco, CA, as opposed to San Diego, CA (3,042 HDD + CDD), is projected to emerge as the city with the lowest combined degree-day sum (2,634 HDD + CDD).

A change in annual degree-day normals may signal a change in corresponding residential heating and cooling demand, as well as outdoor thermal comfort^9^,^28^. By comparing the historical (1981–2010) and projected (2080–2099) degree-day values at several US cities (Table 1), we provide both a qualitative and a quantitative interpretation of the potential effects of global warming on HDD, CDD, and HDD + CDD. For example, in Los Angeles, CA, the historical CDD value (1,247 CDD) is projected to experience about a two-fold increase (2,666 CDD) by the end of the century (2080–2099), resembling the historical CDD value for Jacksonville, FL (2,664 CDD). In contrast, historical HDD value (1,083 HDD) of Los Angeles, CA, is anticipated to decrease (309 HDD), becoming closer to the HDD value of present Miami, FL (128 HDD). The historical combined degree-day value in Los Angeles, CA, (2,330 HDD + CDD) is not expected to increase greatly: by the end of the century, the city’s degree-day sum (2,974 HDD + CDD) is projected to be approximately equal to that of present San Francisco, CA (2,816 HDD + CDD). However, San Francisco’s historical demand is primarily for heating, whereas the demand projected for end-of-century Los Angeles is primarily for cooling. In New York City, NY, an analogous warming effect is anticipated: The historical CDD value of New York City (1,105 CDD) is projected to increase by the end of the century (2,348 CDD), approaching a CDD value that historically prevailed in the hot desert climate of El Paso, TX (2,331 CDD). The historical HDD value (4,750 HDD) in New York City is projected to decrease (3,126 HDD) to approximately the number of HDD in present Raleigh, NC (3,246 HDD). New York City’s historical degree-day sum (5,855 HDD + CDD) will decrease (5,474 HDD + CDD), resembling the historical degree-day sum in Oklahoma City, OK (5,463 HDD + CDD).

Some indication of the reliability of the model projections can be gleaned by comparing historical HDD and CDD values as estimated^31^,^32^ by NOAA for 1981 to 2010 with the model projections interpolated to these same cities for the same years. NOAA provides estimates for 42 of the 50 largest cities in the United States. The root-mean-square (rms) difference between CDD values estimated from observations versus model results for the historical period for these cities is 382 degree-days; for HDD values, the rms difference is 586 degree days. The rms difference in CDD values between modelled future and historical climates for these cities is 1587 degree-days; the corresponding value for HDD is 1715 degree-days. Thus, projected changes in degree-days are much larger than model differences between model results and observations for the historical period. Nevertheless caution should be exercised when interpreting results of relatively coarse-resolution climate models at relatively fine spatial scales.

## Discussion

As rising temperatures^6^ begin to alter weather-related energy demand^4^,^5^,^7^,^14^ in various regions of the US, understanding how heating and cooling demand, as well outdoor thermal comfort, will shift by the end of the century (2080–2099) becomes increasingly important. Here, we investigate how the distribution of NOAA’s^31^,^32^ historical (1981–2010) HDD and CDD normals calculated for 7,438 weather stations in the US would be expected to change in a “business-as-usual” high greenhouse gas emission climate scenario RCP8.5^34^. We use the sum of HDD and CDD normals as an indicator of historical (1981–2010) and future (2080–2099) overall demand for heating and cooling in various US regions and of outdoor thermal comfort. Although studies have previously utilized the degree-day sum to assess the differences in the historical combined heating and cooling demand per individual^10^,^19^, analyses have not been performed for the future degree-day sum in context of global warming. Because the relationship between HDD, CDD, HDD + CDD and energy demand is complex and depends on multiple factors outside the scope of this study (e.g. building design, internal heat sources, insulation)^9^,^10^, we focus on providing an indication, not a robust estimation, of heating and cooling demand in residential buildings. Similar caveats relate to using these values as indicators of outdoor thermal comfort.

We found that, in the historical period (1981–2010), the southern California coast had the minimum combined HDD + CDD values in the contiguous US, and that the coastal Hawaii had slightly smaller values (Fig. 2a). In the future period (2080–2099), however, the coastal California is projected to have the minimum heating and cooling demand (less than in Hawaii), but this minimum region is anticipated to expand northward to the San Francisco region (Fig. 2b). In the contiguous US and Alaska, median combined heating and cooling demand decreases because reductions in heating demand exceed increases in cooling demand. In contrast, this combined heating and cooling demand increases in tropical Hawaii due to increased cooling demand.

In the reference climate change scenario, HDD decreases and CDD increases (Fig. 5a,b), suggesting that heating demand will decline and cooling demand will grow in every region of the US. The greatest increase in CDD values is projected to occur in southern regions, while the largest decrease in HDD values is expected to occur in the northern regions. Overall, the reduction in HDD in the contiguous US will outbalance the increases in CDD. There will be a line, stretching from California to Maryland, across the contiguous US, where the decrease in HDD will approximately equal the increase in CDD, resulting in zero change in the degree-day sum, HDD + CDD (Fig. 5c). While this line is an indicator of minimum change in combined heating and cooling demand, it does not directly relate to the change in the costs of primary and delivered energy.

Some sense of the magnitude of climate change projected for this century can be gleaned by comparing historical and projected HDD values and CDD values for various cities (Table 1 and Supplementary Table S2). For this analysis, we consider HDD and CDD values as two dimensions. For example, if we look at how the HDD values and CDD values of cities in the future period (2080–2099) will be to cities in the historical period (1981–2010) in terms of root-mean-square difference of HDD values and CDD 3568 values (Supplementary Table S2), we find that the projected HDD values and CDD values of the region extending from New York, NY, (HDD 3126; CDD 2348) to Washington, DC, (HDD 2786; CDD 2781) becomes more like that of today’s Las Vegas, NV (HDD 1951; CDD 3568) Boston, MA, becomes similar to today’s Tulsa, OK; Milwaukee, MN, becomes like today’s Wichita, KS. Denver, CO, becomes like Raleigh, NC, and Colorado Springs, CO, becomes like today’s Washington, DC. Las Vegas, NV, becomes like Mesa, AZ, and New York, NY, becomes more like Oklahoma, OK. On the west coast, Seattle, WA, becomes like today’s San Jose, CA, and Portland, OR, becomes like today’s Sacramento, CA. Sacramento becomes similar to Jacksonville, FL. The future San Francisco, CA, is projected to become similar to today’s Los Angeles, CA. Because HDD and CDD were weighted equally, our results do not directly correspond to the differences in price per million of delivered Btu or primary energy for heating and cooling, determined in other studies^5^,^20^,^26^,^27^. Nevertheless, our findings provide relevant metrics indicating changes in heating and cooling demand in the US. Our results could be potentially useful for assisting residents with the selection of housing locations in the present and future, taking into consideration potential effects of global warming on residential building heating and cooling demand and associated outdoor thermal comfort.

## Conclusion

In this work, we analyse the distribution of heating and cooling degree-day sums^10^,^19^ (HDD + CDD) for the United States in the recent historical period of years 1981 to 2010. We furthermore project changes to heating degree-days (HDD), cooling degree-days (CDD), and HDD + CDD for the United States based on CMIP5 projections^33^ of climate change^6^ under the “business as usual” RCP8.5 scenario^34^. Degree-day measures are key indicators of heating and cooling demand^5^,^9^,^10^,^11^,^14^ and outdoor thermal comfort^9^, but other factors such as amount of sunshine, humidity, wind, and characteristics of the built environment also contribute^9^ substantially to actual heating and cooling demand and perceived comfort.

Large CDD values indicate areas with high air conditioning demand or where outdoor climates may at times be uncomfortably warm^9^. Our basic conclusion, with respect to changes in CDD in the United States by the end of this century under a “business-as-usual” climate change scenario, is that areas that currently have high CDD values (e.g. parts of Texas) will have relatively large increases in CDD values, whereas areas with smaller CDD values (e.g. Minnesota) will experience relatively modest increases in CDD values. We project that north-western Washington state will have the smallest increase in CDD values in the United States, whereas Southern Texas will have the greatest increase in CDD values (Fig. 5a).

Large HDD values indicate areas with high heating demand or where outdoor climates may at times be uncomfortably cold^9^. Our basic conclusion, with respect to changes in HDD values in the United States by the end of this century under a “business-as-usual” climate change scenario, is that areas that currently have high HDD values (e.g. North Dakota, Minnesota, and Maine) will have relatively large decreases in HDD values, whereas areas with smaller HDD values (e.g. Florida) will experience relatively modest decreases in HDD values. We project that southern Florida will have the smallest decrease in HDD values while upper North Dakota, Minnesota, and Maine will have the greatest reduction in HDD values (Fig. 5b).

The combined HDD + CDD measure is an indicator of the total amount of heating and cooling demand^11^ and overall outdoor thermal comfort^9^. In the recent historical period, San Diego is the major city with the lowest^8^,^10^ combined HDD + CDD values (1,946 HDD + CDD) (Table 1). However, under a “business-as-usual” climate change scenario, the minimum HDD + CDD city is projected to shift from present San Diego, CA, to future San Francisco, CA (2,634 HDD + CDD). In terms of similarity of HDD values and CDD values, under the RCP8.5 “business as usual” climate scenario, future New York, NY, is projected to have HDD values and CDD values that are similar to those of present Oklahoma City, OK. Using similarity of HDD values and CDD values as the metric for comparison, by the end of this century under this “business as usual” climate change scenario, HDD values and CDD values for Seattle, WA, are projected to become similar to those of present San Jose, CA.

HDD and CDD values are imperfect indicators of heating and cooling demand^9^,^11^,^14^, and of outdoor thermal comfort. Nevertheless, projections such as those presented here should help people to understand the scale of projected climate changes in terms that could influence decisions not only about where to live and work, but also about what energy systems to utilize and what buildings to construct.

## Methods

### Units and symbols

All of the measures used here (HDD, CDD, and HDD + CDD) for the United States have non-SI units of °F × days, equivalent to 4.8 × 10^4^ K s in SI units. We designate this amount using the symbols HDD, CDD or HDD + CDD. We use ΔCDD, ΔHDD, and ΔCDD + ΔHDD to refer to changes in CDD, HDD, or CDD + HDD in the 2080–2099 relative to the 1981–2010 period.

### Base temperature

Historical (1981–2010) and future (2080–2090) absolute degree-days (HDD, CDD, and HDD + CDD), as well as delta degree-days (ΔHDD, ΔCDD, and ΔHDD + ΔCDD) here represented in maps and city tables were calculated for the base temperature of 65 °F (18.3 °C), which is an understandable US standard commonly used in degree-day studies^3^,^9^,^10^,^11^,^18^,^19^,^20^,^26^. The base temperature, however, is not a constant, because it varies among different countries: in South Korea the value ranges^36^ from 14.7 °C to 19.4 °C; in Spain^5^ it is within 15 °C to 17 °C; and in Norway it is set^27^ at 17 °C.

### Historical (1981–2010) NOAA 30-year annual degree-day normals

Daily high-precision HDD normals and CDD normals, computed from temperature observations at 7,438 stations in the contiguous US, Hawaii, and Alaska (see Supplementary Figure S1 and S2), were obtained from NOAA’s 1981–2010 US Climate Normals^31^ dataset (http://www.nature.com/srep/2013/130214/srep01277/full/srep01277.html). NOAA methodology used to compute degree-day normals (i.e. 30-year averages) was previously detailed in Arguez *et al.*^32^. HDD and CDD datasets computed to the standard US base temperature of 65 °F^9^,^10^ (18.3 °C) were chosen. Attribute station data was converted into a table and downloaded to a MySQL database for processing. Annual HDD, CDD, and HDD + CDD normals were calculated by adding respective daily historical (1981–2010) HDD, CDD, or HDD + CDD normals at each station location.

### Historical (1981–2010) and future (2080–2099) CMIP5 annual degree-day normals

Daily minimum and maximum near-surface air temperature variables downloaded from http://pcmdi9.llnl.gov/on 30 June 2014 were obtained for 28 available state-of-the-art climate models (for a full list, see Supplementary Table S1) in the CMIP5 multi-model ensemble archive^34^ for two separate experiments: 1) historical (1850–2005), with all forcings and 2) RCP8.5^35^ (2006–2100), with standardized radiative forcing reaching 8.5 Wm^−2^ in 2100. The RCP8.5 forcing was chosen to represent the “business-as-usual” highest energy demand and green house gas emissions scenario, unmitigated by climate change policies. Absolute daily HDD, CDD, and HDD + CDD simulated by individual models were determined for three time periods (1981–2005; 2005–2010, and 2080–2099) using the UK Meteorological Office equations^9^:


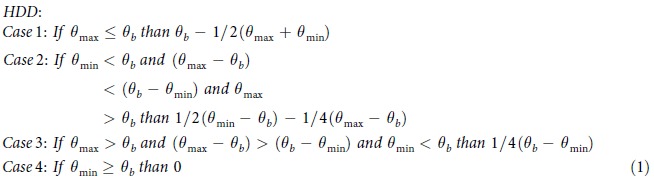



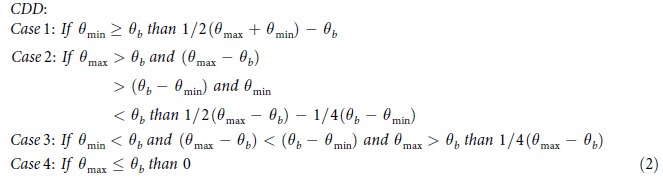


where, in both cases, *θ*_*b*_ is the base temperature of 65 °F (18.3 °C), *θ*_*min*_ is the daily minimum and *θ*_*max*_is the daily maximum near-surface air temperatures, respectively. Although the simplified mean daily temperature method^9^ is standardly used in the US (±5.2%), the UK Met Office approach^9^ was employed here, because it tends to have a smaller expected uncertainty^9^ (±4%). Annual HDD, CDD, and HDD + CDD normals were computed by averaging corresponding absolute daily degree-days at each grid point and later multiplying these values by the number of days in a year. Historical (1981–2010) annual degree-day normals, however, were obtained by additionally calculating the weighted sum of present (1981–2005) and projected (2006–2010) degree-day annual normals. ΔHDD, ΔCDD, and ΔHDD + ΔCDD were computed by subtracting CMIP5 historical (1981–2010) annual degree-day normals from the long-term (2080–2099) projected annual degree-day normals.

### CMIP5 multi-model median and bilinear interpolation

In order to interpolate Δ degree-days projected by CMIP5 models to the coordinates of NOAA stations, an extension of bilinear interpolation equation was used (see Supplementary Equation S1). We assumed that every NOAA station is bounded by the four corners of a rectangle, formed by longitude and latitude of the model’s grid data. All values bounded by the four corners had to follow the formula^37^:


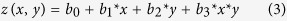


where *z* is the interpolated Δ degree-day value, *b*_*0*_*, b*_*1*_*, b*_*2*_, and *b*_*3*_ are the coordinates of the four corners of a rectangle, and *(x, y)* is the coordinate of a station. A median across all CMIP5 models used in the study was found for each Δ degree-day value at a particular station location. Annual HDD, CDD, and HDD + CDD normals for the future (2080–2099) period were found by adding the corresponding projected ΔHDD, ΔCDD, and ΔHDD + ΔCDD interpolated to NOAA stations to the historical NOAA degree-day normals.

### Graphic software

All maps were produced by separately interpolating degree-day normals for the contiguous US, Alaska, and Hawaii, using licensed ArcMap 10.2 Geostatistical Analyst tool for ordinary kriging. Station median degree-day values were obtained from embedded surface summary statistics. City tables were created in licensed Microsoft Excel.

### Degree-days at the 25 most populous US incorporated places

Historical (1981–2010) and long-term (2080–2099) annual HDD, CDD, and HDD + CDD normals were tabulated for 50 most populous incorporated places^35^ provided by the United States Census Bureau in the latest Vintage 2013. Historical (1981–2010) HDD (July–June) and CDD (January–December) normals are 30-year annual degree-day averages at major weather stations in each of the chosen cities included in the NOAA Comparative Climatic Data publication^38^. If more than one station was used to represent degree-day normals at a selected city, the degree-day value at the city’s major airport was recorded. Future (2080–2099) annual HDD and CDD normals were computed from the median CMIP5 multi-model degree-day output interpolated to city point observation locations using ArcMap 10.2 default cities layer and GA layer to points tool. Balances and metropolitan governments were assigned the degree-day value of their largest included city. Seven of the 25 selected urban areas (i.e. San Francisco, Phoenix, Miami, Minneapolis, San Diego, Seattle, and Mesa) had the lowest or highest degree-day index in either of the two time periods; four of the chosen areas (i.e. New York, Los Angeles, Chicago, and Houston) were the most populous on the list (2,000,000+), and the rest of the 14 areas were included for the purposes of comparison.

## Additional Information

**How to cite this article**: Petri, Y. and Caldeira, K. Impacts of global warming on residential heating and cooling degree-days in the United States. *Sci. Rep.* **5**, 12427; doi: 10.1038/srep12427 (2015).

## Supplementary Material

Supplementary Information

## Figures and Tables

**Table 1:** Historical (1981–2010) and future (2080–2099) annual HDD, CDD, and HDD + CDD normals tabulated for 25 selected cities from the list of 50 most populous incorporated places provided by the United States Census Bureau in the latest Vintage 2013^35^.

		NOAA Comparative Climatic Data (1981–2010)			Projected Long-term (2080–2099) RCP8.5 Scenario		
Rank	Incorporated Places of 50,000 or More	HDD	CDD	HDD + CDD	HDD	CDD	HDD + CDD
1	San Francisco city, CA	2,653	163	2,816	1,410	1,224	2,634
2	Los Angeles city, CA	1,083	1,247	2,330	309	2,666	2,974
3	San Diego city, CA	1,226	720	1,946	495	2,547	3,042
4	Seattle city, WA	4,370	188	4,558	2,576	898	3,473
5	El Paso city, TX	2,473	2,331	4,804	1,092	3,609	4,701
6	Albuquerque city, NM	4,180	1,322	5,502	2,824	2,032	4,856
7	Denver city, CO	6,059	769	6,828	3,504	1,545	5,049
8	Las Vegas city, NV	1,951	3,568	5,519	1,140	4,021	5,161
9	Atlanta city, GA	2,767	1,882	4,649	1,956	3,306	5,262
10	Phoenix city, AZ	935	4,608	5,543	9	5,425	5,435
11	Jacksonville city, FL	1,350	2,664	4,014	757	4,693	5,451
12	New York city, NY	4,750	1,105	5,855	3,126	2,348	5,474
13	Mesa city^a^, AZ	1,350	3,720	5,070	0^b^	5,481	5,481
14	Raleigh city, NC	3,246	1,731	4,977	2,224	3,276	5,500
15	Houston city, TX	1,289	2,939	4,228	733	4,896	5,628
16	Philadelphia city, PA	4,612	1,301	5,913	3,124	2,508	5,632
17	Boston city, MA	5,681	747	6,428	3,985	1,715	5,700
18	Memphis city, TN	2,965	2,258	5,223	2,041	3,744	5,784
19	Tulsa city, OK	3,693	1,915	5,608	2,267	3,654	5,921
20	Oklahoma City city, OK	3,365	2,098	5,463	2,362	3,750	6,112
21	Detroit city, MI	6,168	824	6,992	4,222	1,915	6,137
22	Chicago city, IL	6,339	842	7,181	4,059	2,217	6,276
23	Miami city, FL	128	4,575	4,703	92	6,360	6,452
24	Wichita city, KS	4,592	1,686	6,278	3,186	3,274	6,460
25	Minneapolis city, MN	7,581	752	8,333	5,084	2,090	7,174

Columns are arranged in order of increasing future (2080–2099) HDD + CDD normals. Projected “HDD” and “CDD” do not always add up to “HDD + CDD” due to rounding.

^a^Historical HDD and CDD for this city were not included into the NOAA Comparative Climatic Data publication^38^. Annual degree-day normals were calculated by interpolating historical (1981–2010) degree-day normals recorded at NOAA meteorological stations to the city’s longitude and latitude obtained from the ArcMap 10.2 default point cities layer.

^b^Formal mathematics produced a negative CMIP5 projected (2080–2099) HDD normal for Mesa, AZ. This value likely represents an interpolation error caused by the city’s proximity to locations of weather stations for which CMIP5 projected (2080–2099) HDD normals were close or equal to zero. Because degree-days cannot be negative, the misleading value was replaced by 0 in Table 1.
