# Supplementary material for: Impacts of global warming on residential heating and cooling degree-days in the United States
Source: Sci Rep. 2015 Aug 4;5:12427. doi: 10.1038/srep12427 (PMC4523835; doi:10.1038/srep12427)
Supplement: Supplementary Information [file srep12427-s1.pdf]

Supplementary information for "Impacts of global warming on residential heating and cooling degree-days in the United States"

Yana Petri[1] & Ken Caldeira[1*]

[1]Carnegie Institution for Science, Department of Global Ecology, Stanford, CA, USA

*Correspondence and requests for materials should be addressed to KC (kcaldeira@carnegiescience.edu)

Supplementary Table S1: 28 CMIP5 models and institutions that provided minimum and maximum near-surface air-temperature output used to project degree-days in this study. Each utilized atomic dataset, except for HadGEM2-ES (r3), had the first realization number (r1).

#	Model Name(s)	Institution(s)
1	ACCESS1.0	Commonwealth Scientific and Industrial Research Organization (CSIRO) and Bureau of Meteorology (BOM), Australia
2	ACCESS1.3	
3	BCC-CSM1.1	Beijing Climate Center, China Meteorological Administration
4	BCC-CSM1.1(m)	
5	BNU-ESM	College of Global Change and Earth System Science, Beijing Normal University
6	CanESM2	Canadian Centre for Climate Modelling and Analysis
7	CCSM4	
8	CESM1(BGC)	Community Earth System Model Contributors
9	CMCC-CESM	Centro Euro-Mediterraneo per I Cambiamenti Climatici
10	CMCC-CM	
11	CMCC-CMS	
12	CNRM-CM5	Centre National de Recherches Météorologiques / Centre Européen de Recherche et Formation Avancée en Calcul Scientifique
13	CSIRO-Mk3.6.0	Commonwealth Scientific and Industrial Research Organization in collaboration with Queensland Climate Change Centre of Excellence
14	FGOALS-g2	LASG, Institute of Atmospheric Physics, Chinese Academy of Sciences and CESS, Tsinghua University
15	GFDL-CM3	NOAA Geophysical Fluid Dynamics Laboratory
16	GFDL-ESM2G	
17	GFDL-ESM2M	
18	HadGEM2-CC	Met Office Hadley Centre (additional HadGEM2-ES realizations contributed by Instituto Nacional de Pesquisas Espaciais)
19	HadGEM2-ES	
20	INM-CM4	Institute for Numerical Mathematics
21	IPSL-CM5A-LR	Institut Pierre-Simon Laplace
22	IPSL-CM5A-MR	
23	IPSL-CM5B-LR	
24	MIROC-ESM	Japan Agency for Marine-Earth Science and Technology, Atmosphere and Ocean Research Institute (The University of Tokyo), and National Institute for Environmental Studies
25	MIROC-ESM-CHEM	
26	MIROC5	Atmosphere and Ocean Research Institute (The University of Tokyo), National Institute for Environmental Studies, and Japan Agency for Marine-Earth Science and Technology
27	MRI-CGCM3	Meteorological Research Institute
28	NorESM1-M	Norwegian Climate Centre

Supplementary Table S2: Historical (1981–2010), near-term (2016-2035), and long-term (2080–2099) annual HDD, CDD, and HDD+CDD normals tabulated for 50 cities from the list of the most populous incorporated places[1] of 50,000 and more provided by the United States Census Bureau in the latest Vintage 2013. Columns are arranged in order of increasing projected long-term (2080–2099) annual HDD+CDD normals.

		NOAA Comparative Climatic Data (1981–2010)			Projected Near-term (2016–2035) RCP8.5 Scenario			Projected Long-term (2080–2099) RCP8.5 Scenario		
Rank	Incorporated Places of 50,000 or More	HDD	CDD	HDD+CDD	HDD	CDD	HDD+CDD	HDD	CDD	HDD+CDD
1	San Francisco city, CA	2,653	163	2,816	2,514	397	2,911	1,410	1,224	2,634
2	Oakland city[a], CA	2,681	498	3,179	2,277	686	2,963	1,159	1,511	2,670
3	Long Beach city, CA	1,217	1,126	2,343	1,104	1,312	2,416	251	2,552	2,803
4	San Jose city, CA	2,707	601	3,308	2,311	795	3,105	1,221	1,660	2,881
5	Los Angeles city, CA	1,083	1,247	2,330	1,204	1,442	2,646	309	2,666	2,974
6	San Diego city, CA	1,226	720	1,946	1,259	1,250	2,509	495	2,547	3,042
7	Seattle city, WA	4,370	188	4,558	4,277	260	4,537	2,576	898	3,473
8	Sacramento city, CA	2,618	1,178	3,796	2,257	1,559	3,815	1,010	2,490	3,500
9	Portland city, OR	4,277	424	4,701	4,004	440	4,444	2,506	1,020	3,526
10	Fresno city, CA	2,346	2,124	4,470	2,688	1,729	4,417	1,488	2,818	4,306
11	El Paso city, TX	2,473	2,331	4,804	2,363	2,322	4,684	1,092	3,609	4,701
12	Albuquerque city, NM	4,180	1,322	5,502	4,475	1,056	5,530	2,824	2,032	4,856
13	Tucson city, AZ	1,537	3,072	4,609	1,592	2,985	4,576	555	4,442	4,997
14	Denver city, CO	6,059	769	6,828	5,377	783	6,160	3,504	1,545	5,049
15	Las Vegas city, NV	1,951	3,568	5,519	2,539	2,757	5,296	1,140	4,021	5,161
16	Atlanta city, GA	2,767	1,882	4,649	2,825	1,956	4,781	1,956	3,306	5,262
17	Charlotte city, NC	3,390	1,519	4,909	3,099	1,929	5,028	2,079	3,246	5,324
18	Virginia Beach city[a], VA	3,346	1,590	4,936	3,074	1,934	5,008	2,186	3,239	5,425
19	Phoenix city, AZ	935	4,608	5,543	1,112	3,989	5,101	9	5,425	5,435
20	Jacksonville city, FL	1,350	2,664	4,014	1,131	3,094	4,226	757	4,693	5,451
21	New York city, NY	4,750	1,105	5,855	4,613	1,268	5,880	3,126	2,348	5,474
22	Mesa city[a], AZ	1,350	3,720	5,070	980	4,098	5,078	0[b]	5,481	5,481
23	Raleigh city, NC	3,246	1,731	4,977	3,214	1,898	5,112	2,224	3,276	5,500
24	Washington city, DC	4,717	1,178	5,895	4,127	1,560	5,687	2,786	2,781	5,567
25	Baltimore city, MD	4,764	1,164	5,928	4,240	1,531	5,771	2,850	2,730	5,580
26	Houston city, TX	1,289	2,939	4,228	1,240	3,353	4,594	733	4,896	5,628
27	Philadelphia city, PA	4,612	1,301	5,913	4,576	1,371	5,947	3,124	2,508	5,632
28	Nashville-Davidson metropolitan government (balance), TN	3,688	1,647	5,335	3,652	1,819	5,470	2,573	3,116	5,689
29	Boston city, MA	5,681	747	6,428	5,645	818	6,463	3,985	1,715	5,700
30	Colorado Springs city, CO	6,292	455	6,747	6,238	563	6,801	4,380	1,368	5,747
31	Louisville/Jefferson County metro government (balance), KY	4,269	1,444	5,713	4,268	1,547	5,815	2,960	2,798	5,758
32	Austin city, TX	1,540	3,140	4,680	1,631	3,193	4,823	965	4,800	5,765
33	Fort Worth city, TX	2,286	2,755	5,041	2,129	3,022	5,152	1,303	4,466	5,770
34	Arlington city[a], TX	2,374	2,599	4,974	2,062	3,081	5,142	1,244	4,526	5,770
35	Cleveland city, OH	5,761	817	6,578	2,572	2,569	5,141	1,721	4,057	5,778
36	San Antonio city, TX	1,410	3,187	4,597	1,336	3,407	4,743	707	5,073	5,779
37	Memphis city, TN	2,965	2,258	5,223	3,009	2,350	5,359	2,041	3,744	5,784
38	Dallas city, TX	2,286	2,755	5,041	2,154	3,012	5,166	1,331	4,466	5,797
39	Tulsa city, OK	3,693	1,915	5,608	4,370	1,946	6,315	2,267	3,654	5,921
40	Columbus city, OH	5,250	1,034	6,284	5,191	1,182	6,373	3,638	2,330	5,968
41	Oklahoma City city, OK	3,365	2,098	5,463	3,365	2,362	5,728	2,362	3,750	6,112
42	Detroit city, MI	6,168	824	6,992	6,021	913	6,934	4,222	1,915	6,137
43	Indianapolis city (balance), IN	5,348	1,059	6,407	5,272	1,260	6,532	3,805	2,451	6,256
44	Chicago city, IL	6,339	842	7,181	5,777	1,147	6,924	4,059	2,217	6,276
45	Kansas City city, MO	4,686	1,673	6,359	4,666	1,758	6,424	3,334	3,033	6,367
46	Miami city, FL	128	4,575	4,703	134	4,697	4,831	92	6,360	6,452
47	Wichita city, KS	4,592	1,686	6,278	4,370	1,946	6,315	3,186	3,274	6,460
48	Milwaukee city, WI	6,894	641	7,535	6,601	807	7,409	4,735	1,804	6,539
49	Omaha city, NE	6,506	906	7,412	5,821	1,366	7,187	4,270	2,521	6,790
50	Minneapolis city, MN	7,581	752	8,333	7,074	969	8,043	5,084	2,090	7,174

[a] Historical HDD and CDD for this city were not included into the NOAA Comparative Climatic Data publication[38]. Annual degree-day normals were calculated by interpolating historical (1981–2010) degree-day normals recorded at NOAA meteorological stations to the city's longitude and latitude obtained from the ArcMap 10.2 default point cities layer.

[b] Formal mathematics produced a negative CMIP5 projected (2080-2099) HDD normal for Mesa, AZ. This value likely represents an interpolation error caused by the city's proximity to locations of weather stations for which CMIP5 projected (2080-2099) HDD normals were close or equal to zero. Because degree-days cannot be negative, the misleading value was replaced by 0 in Supplementary Table S2.

Supplementary Table S3: Near-term (2016-2035) and long-term (2080–2099) ΔHDD, ΔCDD, and ΔHDD+ΔCDD tabulated for 50 cities from the list of the most populous incorporated places[1] of 50,000 and more provided by the United States Census Bureau in the latest Vintage 2013. Columns are arranged in order of increasing long-term (2080–2099) ΔHDD+ΔCDD.

		Projected Near-term (2016–2035) RCP8.5 Scenario			Projected Long-term (2080–2099) RCP8.5 Scenario		
Rank	Incorporated Places of 50,000 or More	ΔHDD	ΔCDD	ΔHDD+ΔCDD	ΔHDD	ΔCDD	ΔHDD+ΔCDD
1	Denver city, CO	-669	157	-512	-2,542	913	-1,629
2	Seattle city, WA	-646	125	-521	-2,355	765	-1,590
3	Colorado Springs city, CO	-635	166	-469	-2,488	963	-1,525
4	Portland city, OR	-592	125	-467	-2,091	707	-1,384
5	Minneapolis city, MN	-683	291	-391	-2,677	1,410	-1,266
6	Milwaukee city, WI	-597	270	-327	-2,452	1,257	-1,195
7	Detroit city, MI	-553	250	-302	-2,354	1,243	-1,111
8	Boston city, MA	-534	221	-313	-2,197	1,123	-1,074
9	Albuquerque city, NM	-547	220	-327	-2,197	1,167	-1,029
10	Cleveland city, OH	-523	251	-272	-2,261	1,274	-987
11	Chicago city, IL	-562	315	-247	-2,288	1,370	-917
12	New York city, NY	-449	278	-172	-1,940	1,352	-588
13	Omaha city, NE	-558	374	-185	-2,111	1,526	-585
14	Columbus city, OH	-456	305	-150	-2,011	1,450	-561
15	Sacramento city, CA	-434	223	-211	-1,679	1,167	-512
16	San Francisco city, CA	-402	176	-226	-1,507	1,002	-505
17	Oakland city, CA	-402	191	-211	-1,513	1,035	-478
18	Philadelphia city, PA	-435	300	-135	-1,881	1,438	-443
19	San Jose city, CA	-396	194	-202	-1,484	1,059	-425
20	Indianapolis city (balance)[b] , IN	-458	344	-113	-1,925	1,537	-388
21	Las Vegas city, NV	-448	289	-159	-1,871	1,498	-373
22	Baltimore city, MD	-425	297	-128	-1,813	1,488	-325
23	Fresno city, CA	-422	268	-153	-1,631	1,356	-275
24	Washington city, DC	-408	305	-103	-1,751	1,522	-229
25	Louisville/Jefferson County metro government (balance)[b] , KY	-400	355	-45	-1,709	1,603	-106
26	Kansas City city, MO	-479	441	-38	-1,813	1,713	-100
27	El Paso city, TX	-434	339	-94	-1,701	1,618	-83
28	Wichita city, KS	-447	420	-27	-1,636	1,741	105
29	Nashville-Davidson metropolitan government (balance)[b] , TN	-358	384	26	-1,437	1,680	243
30	Mesa city, AZ	-370	378	8	-1,496	1,767	271
31	Los Angeles city, CA	-324	290	-34	-1,220	1,517	297
32	Phoenix city, AZ	-364	380	15	-1,480	1,781	301
33	Charlotte city, NC	-313	357	44	-1,335	1,675	340
34	Tulsa city, OK	-386	435	49	-1,449	1,800	351
35	Long Beach city, CA	-312	290	-22	-1,164	1,523	360
36	Tucson city, AZ	-370	386	16	-1,408	1,814	406
37	Raleigh city, NC	-295	350	55	-1,292	1,732	440
38	Oklahoma City city, OK	-370	447	77	-1,370	1,828	457
39	Virginia Beach city, VA	-272	343	71	-1,151	1,647	496
40	Memphis city, TN	-337	416	79	-1,306	1,808	502
41	Atlanta city, GA	-281	357	76	-1,148	1,707	559
42	San Diego city, CA	-272	320	48	-1,019	1,598	580
43	Fort Worth city, TX	-316	478	162	-1,144	1,918	774
44	Arlington city, TX	-313	481	167	-1,135	1,924	789
45	Dallas city, TX	-312	482	170	-1,136	1,930	794
46	Austin city, TX	-258	483	225	-924	2,097	1,173
47	San Antonio city, TX	-258	474	216	-889	2,144	1,255
48	Houston city, TX	-211	446	235	-717	1,977	1,260
49	Jacksonville city, FL	-124	423	299	-499	2,021	1,522
50	Miami city, FL	-18	442	423	-61	2,104	2,043

[a] Historical degree-days for this city were not included into the NOAA Comparative Climatic Data publication[2]. Annual degree-day normals were calculated by interpolating historical (1981–2010) degree-day normals recorded at NOAA meteorological stations to the city's longitude and latitude in ArcMap 10.2 default point cities layer.

[b] Historical annual degree-day normals for a balance were approximated by using degree-day normals recorded at the weather station of its largest city.

Supplementary Equation S1: Extension of the bilinear interpolation equation used to interpolate CMIP5 results as described in the Methods section, where the four corners of a rectangle are (x0, y0, z0), (x1, y1, z1), (x2, y2, z3) and (x3, y3, z3), where the x-values represent latitudes, y-values represent longitudes, and the z-values represent the quantity to be regridded.

$$
z = (x2 * x3 * y * y2 * z0 - x2 * x3 * y1 * y2 * z0 - x2 * x3 * y * y3 * z0 + x2 * x3 * y1 * y3 * z0 - x0 * x2 * y * y0 * z1 + x0 * x3 * y * y0 * z1 + x0 * x2 * y * y2 * z1 - x2 * x3 * y * y2 * z1 - x0 * x3 * y0 * y2 * z1 + x2 * x3 * y0 * y2 * z1 - x0 * x3 * y * y3 * z1 + x2 * x3 * y * y3 * z1 + x0 * x2 * y0 * y3 * z1 - x2 * x3 * y0 * y3 * z1 - x0 * x2 * y2 * y3 * z1 + x0 * x3 * y2 * y3 * z1 - x0 * x3 * y * y0 * z2 + x0 * x3 * y0 * y1 * z2 + x0 * x3 * y * y3 * z2 - x0 * x3 * y1 * y3 * z2 + x0 * x2 * y * y0 * z3 - x0 * x2 * y0 * y1 * z3 - x0 * x2 * y * y2 * z3 + x0 * x2 * y1 * y2 * z3 + x1 * (x3 * (y1 - y3) * (y2 * z0 - y0 * z2 + y * (-z0 + z2)) + x2 * (y1 - y2) * (-y3 * z0 + y * (z0 - z3) + y0 * z3) + x0 * (y0 - y1) * (-y3 * z2 + y * (z2 - z3) + y2 * z3)) + x * (x1 * y * y2 * z0 - x1 * y1 * y2 * z0 - x1 * y * y3 * z0 + x1 * y1 * y3 * z0 - x0 * y * y2 * z1 + x0 * y0 * y2 * z1 + x0 * y * y3 * z1 - x0 * y0 * y3 * z1 - x1 * y * y0 * z2 + x0 * y * y1 * z2 - x0 * y0 * y1 * z2 + x1 * y0 * y1 * z2 - x0 * y * y3 * z2 + x1 * y * y3 * z2 + x0 * y0 * y3 * z2 - x1 * y1 * y3 * z2 + x3 * (y - y3) * (y1 * z0 - y2 * z0 - y0 * z1 + y2 * z1 + y0 * z2 - y1 * z2) + x1 * y * y0 * z3 - x0 * y * y1 * z3 + x0 * y0 * y1 * z3 - x1 * y0 * y1 * z3 + x0 * y * y2 * z3 - x1 * y * y2 * z3 - x0 * y0 * y2 * z3 + x1 * y1 * y2 * z3 + x2 * (y - y2) * (y3 * z0 + y0 * z1 - y3 * z1 - y0 * z3 + y1 * (-z0 + z3))))/(x1 * (x2 * (y1 - y2) * (y0 - y3) - x3 * (y0 - y2) * (y1 - y3)) + x0 * (x3 * (y1 - y2) * (y0 - y3) - x2 * (y0 - y2) * (y1 - y3) + x1 * (y0 - y1) * (y2 - y3)) + x2 * x3 * (y0 - y1) * (y2 - y3))
$$

Supplementary Figure S1: Locations of 7,438 weather stations in the contiguous US, Alaska, and Hawaii for which the latest 30-year (1981–2010) daily high-precision degree-day normals were produced by NOAA (National Oceanic and Atmospheric Administration). Historical (1981–2010) annual HDD and CDD calculated for each of the following stations were used to conduct ordinary kriging. The map was generated in ESRI ArcMap 10.2 (Environmental Systems Resource Institute, ArcMap 10.2 ESRI, Redlands, California, USA).

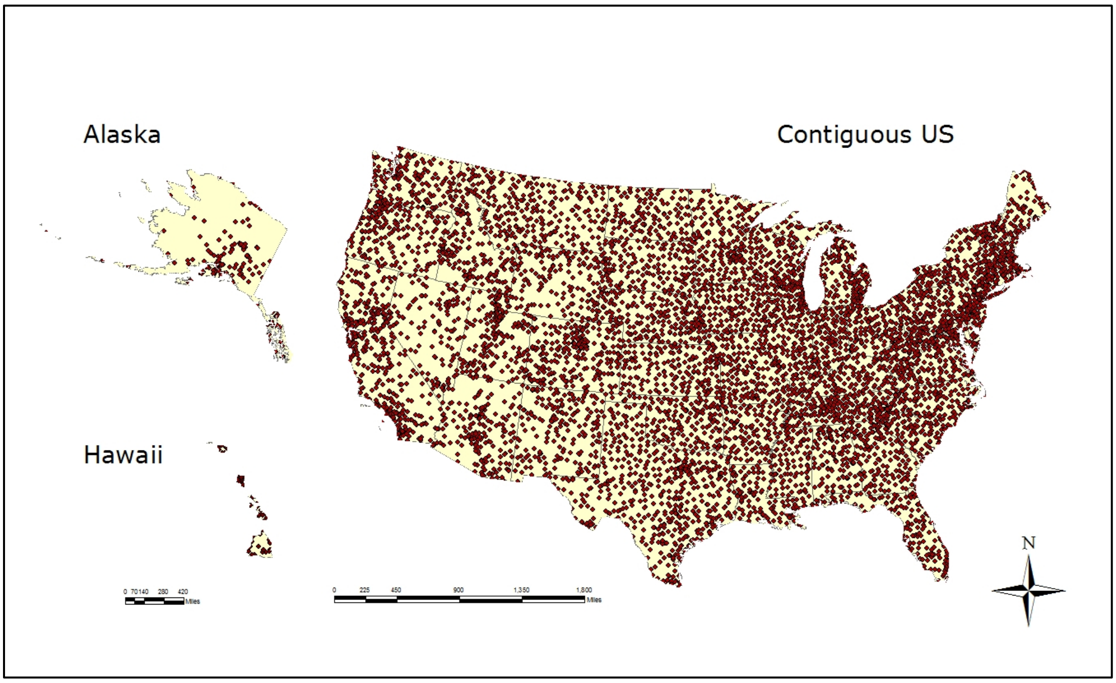

Supplementary Figure S2: Downscaled map represents locations of 392 weather stations in California for which the latest 30-year (1981–2010) daily high-precision degree-day normals were produced by NOAA (National Oceanic and Atmospheric Administration). Historical (1981–2010) annual HDD and CDD calculated for each of the following stations were used to conduct ordinary kriging. The map was generated in ESRI ArcMap 10.2 (Environmental Systems Resource Institute, ArcMap 10.2 ESRI, Redlands, California, USA).

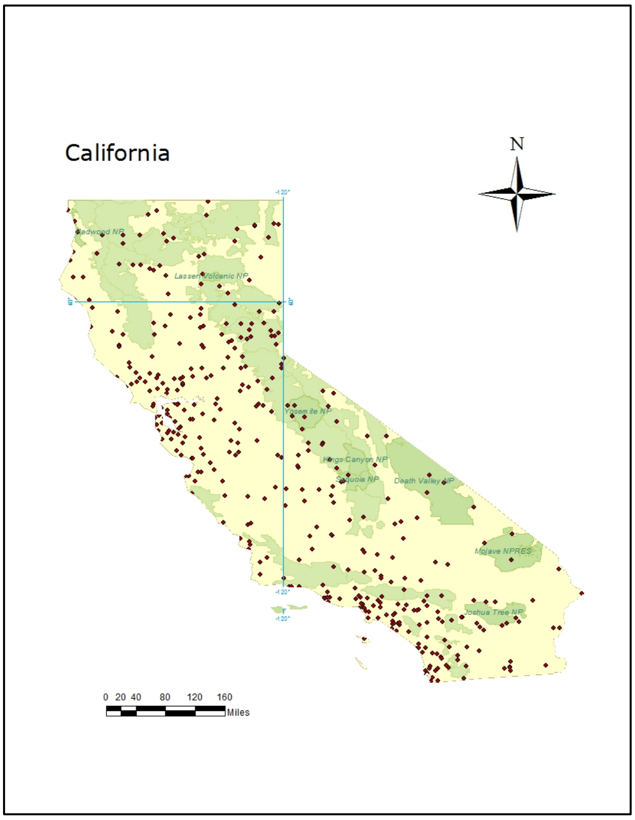

References

1. US Census Bureau, Population Division. Annual estimates of the resident population for incorporated places of 50,000 or more (2014).

2. *Comparative Climatic Data for the United States Through 2012*. (National Climatic Data Center, Asheville, 2013).
